# Nickel chloride-induced apoptosis via mitochondria- and Fas-mediated caspase-dependent pathways in broiler chickens

**DOI:** 10.18632/oncotarget.12946

**Published:** 2016-10-27

**Authors:** Hongrui Guo, Hengmin Cui, Jing Fang, Zhicai Zuo, Junliang Deng, Xun Wang, Ling Zhao, Bangyuan Wu, Kejie Chen, Jie Deng

**Affiliations:** ^1^ College of Veterinary Medicine, Sichuan Agricultural University, Ya'an 625014, China; ^2^ Key Laboratory of Animal Diseases and Environmental Hazards of Sichuan Province, Sichuan Agricultural University, Ya'an 625014, China

**Keywords:** NiCl_2_, apoptosis, mitochondria-mediated caspase-dependent apoptosis, Fas-mediated caspase-dependent apoptosis, kidney

## Abstract

Ni, a metal with industrial and commercial uses, poses a serious hazard to human and animal health. In the present study, we used flow cytometry, immunohistochemistry and qRT-PCR to investigate the mechanisms of NiCl_2_-induced apoptosis in kidney cells. After treating 280 broiler chickens with 0, 300, 600 or 900 mg/kg NiCl_2_ for 42 days, we found that two caspase-dependent pathways were involved in the induced renal tubular cell apoptosis. In the mitochondria-mediated caspase-dependent apoptotic pathway, cyt-c, HtrA2/Omi, Smac/Diablo, apaf-1, PARP, and caspase-9, 3, 6 and 7 were all increased, while. XIAP transcription was decreased. Concurrently, in the Fas-mediated caspase-dependent apoptotic pathway, Fas, FasL, caspase-8, caspase-10 and Bid levels were all increased. These results indicate that dietary NiCl_2_ at 300+ mg/kg induces renal tubular cell apoptosis in broiler chickens, involving both mitochondrial and Fas-mediated caspase-dependent apoptotic pathways. Our results provide novel insight into Ni and Ni-compound toxicology evaluated *in vitro* and *in vivo*.

## INTRODUCTION

Ni is the most abundant element in the earth's crust [1]. Due to its superior heat and electricity conductivity and high melting point [2], Ni and its alloys are widely used as catalysts and pigments across multiple global industries [3]. Widely used Ni salts exploited commercially include nickel chloride, sulphate, hydroxide, acetate, oxide and others.

Accelerated consumption of Ni-containing products induces discharge of Ni-pollutants into the environment. Excessive exposure to Ni may be harmful to human and animal health [4, 5]. Ni is one of the most commonly detected cutaneous allergens in children in the United States [6]. Epidemiological studies have associated Ni exposure with increased risk of nasal and lung cancer [2, 7], and the International Agency for Research on Cancer has classified Ni as an important human carcinogen [8]. Ni can accumulate in kidney, lung, bone, liver and heart, and exposure to Ni or Ni compounds can induce organ system-toxicity [9–12]. Gathwan, *et al.* [13] suggested that NiCl_2_ can induce hepatic DNA damage in mice. NiSO_4_ can induce apoptosis and oxidative stress in rat testes [14] and mouse liver [15]. The percentage of apoptotic cells is increased in porcine granulosa cells after exposure to 1,000 μmol/L NiCl_2_ [16]. Our previous findings also showed that dietary NiCl_2_ at 300+ mg/kg induces immunotoxicity, oxidative stress, apoptosis and cell cycle arrest in the kidney, spleen, small intestines, cecal tonsil and bursa of Fabricius of broiler chickens [17–25].

Apoptosis, or programmed cell death, plays a major role in homeostasis and development in multi-cellular organisms [26]. The caspase cascade promotes induction, transduction and amplification of intracellular apoptotic signaling [27, 28]. In the apoptotic pathway, caspases can be divided into initiators and executioners. The “initiator caspases” include caspase-2, 8, 9, 10 and 12, which are closely coupled to upstream pro-apoptotic signals. Initiator caspases cleave and activate “executioner” caspases, including caspase-3, 6 and 7, which ultimately induce apoptosis [29]. NiONPs increase caspase-3 activation and apoptosis rates in human bronchial epithelial cells [30]. Nickel acetate increases cyt-c and caspase-9, 3 and 6 protein levels in human proximal tubule cells [31]. Buschini, *et al.* [32] reported that exposure of p53-defective human leukemia cells (U937) to bis(S-citronellalthiosemicarbazonato)nickel(II) (Ni(tcitr)_2_) causes apoptosis via Bcl-2 downregulation, disruption of MMP and increased caspase-3 activity. Thus far, only Zhao, *et al.* [33] suggested that metallic nickel particles can induce Fas-mediated apoptosis in JB6 cells.

The precise mechanisms involved in Ni and Ni compound-induced apoptosis are presently still unclear. Mitochondria- and Fas-mediated caspase-dependent pathways are the main apoptosis regulatory mechanisms. Thus far, there have been no *in vitro* or *in vivo* systematic studies of mitochondria- and Fas-mediated caspase-dependent apoptosis induced by Ni and Ni compounds. The purpose of the present study was to investigate whether NiCl_2_ induced apoptosis in the broiler chicken kidney via caspase-dependent pathways. This study was designed to monitor apoptosis in the kidney and to elucidate possible NiCl_2_-induced apoptosis mechanisms, including mitochondria- and Fas-mediated caspase-dependent pathways.

## RESULTS

### Pathological changes in animals

Clinical observations were performed as previously described [34]. From 14 to 42 d, broiler feed intake in the three NiCl_2_-treated groups, except the 300 mg/kg group at 14 d, began to decline compared to controls, From 21 to 42 d, broilers in the three NiCl_2_-treated groups exhibited signs of depression and showed reduced appetites and growth. A few broilers showed polypnea. No unexpected deaths occurred during the experiment.

**Figure 1:**
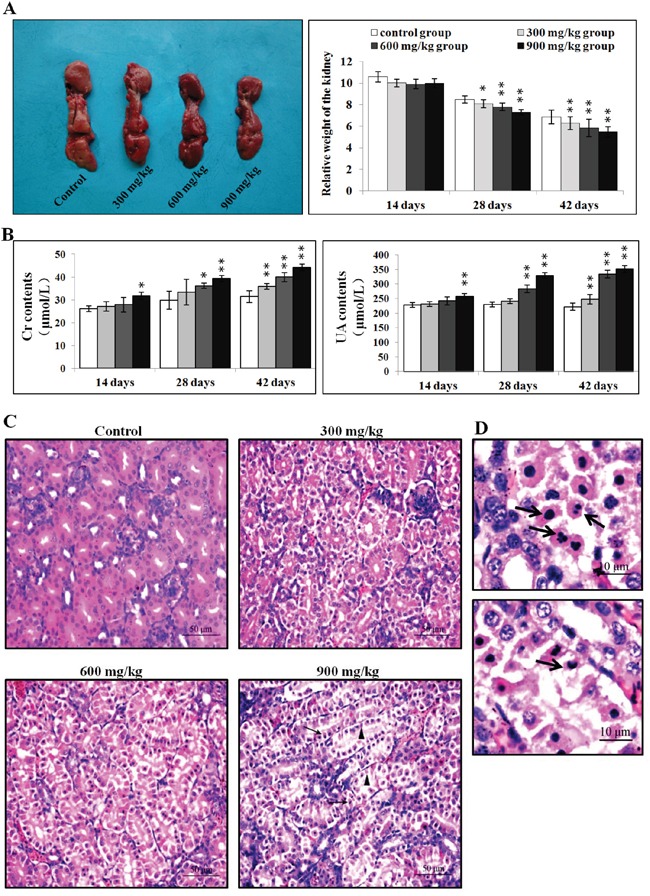
Pathological changes in the kidney **A.** Changes in kidney size and relative weight at 42 d. **B.** Changes in serum creatinine and uric acid levels. **C.** Histopathological changes in the kidney at 42 d. Control group: no changes observed; 300 mg/kg group: tubular cells show granular and vacuolar degeneration, few necrotic tubular cells and apoptotic tubular cells are observed; 600 mg/kg group: tubular cells show marked granular and vacuolar degeneration, some necrotic tubular cells and apoptotic tubular cells are observed; 900 mg/kg group: Many necrotic tubular cells (▲) and apoptotic tubular cells (↑) are observed. (H·E ×400). **D.** Morphological changes in apoptotic cells. Apoptotic cell cytoplasm was intensely eosinophilic, and nuclei were shrunken, dense, ring-shaped or crescentic. Some apoptotic cell nuclei were cracked into two or multiple apoptotic bodies (↑). (H·E ×1000). Data are presented as means ± standard deviation (n=5). *P<0.05, **P<0.01 compared with the control group.

There were no macroscopic changes in the three NiCl_2_-treated groups during the experiment compared to controls. However, relative kidney weights were lower (P<0.05 or P<0.01) in the three NiCl_2_-treated groups than in the control group at 28 and 42 d (Figure 1A).

### Creatinine and uric acid levels

Serum creatinine and uric acid levels were higher (P<0.05 or P<0.01) in the 900 mg/kg group at 14 d, in the 600 and 900 mg/kg groups from 28 to 42 d, and in the 300 mg/kg group at 42 d as compared to the control group (Figure 1B).

### Histopathological changes

NiCl_2_ induced dose- and time-dependent histopathological changes in the kidney, including tubular granular degeneration, vacuolar degeneration, necrosis and apoptosis. Small particles and variably-sized vacuoles appeared in the cytoplasm of degenerated cells (Figure 1C) [20]. Necrotic cells exhibited karyorrhexis, karyolysis and hypochromatosis. The cytoplasm of apoptotic cells was eosinophilic (Figure 1D) [20]. Nuclei were shrunken, dense, ring-shaped and crescentic. Apoptotic bodies were observed.

### NiCl_2_ increased apoptosis in the kidney

**Figure 2:**
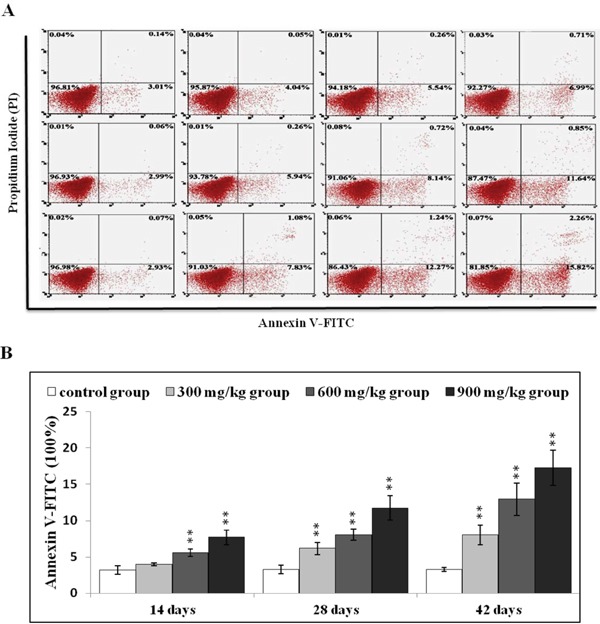
NiCl_2_ induces apoptosis in the kidney **A.** Representative flow cytometry apoptosis analysis diagram. **B.** Changes in numbers of annexin V positive cells in the kidney Data are presented with the mean ± standard deviation (n=5). *P<0.05, **P<0.01 compared with the control group.

After NiCl_2_ treatment, renal cells were labeled using Annexin V-FITC and PI to discriminate live (Annexin V-FITC^−^ and PI^−^), early apoptotic (Annexin V-FITC^+^ and PI^−^), late apoptotic (Annexin V-FITC^+^ and PI^+^) and primary/secondary necrotic cells (Annexin V-FITC^−^ and PI^+^). Apoptotic cells (early apoptotic + late apoptotic cells) were more prevalent in the 600 mg/kg and 900 mg/kg groups at 14 d compared to the control group (P<0.05 or P<0.01) (Figure 2), and increased in the three NiCl_2_-treated groups from 28 to 42 d (P<0.05 or P<0.01).

### Mitochondria-mediated caspase-dependent apoptosis

In our previous study, NiCl_2_ disrupted MMP and increased AIF and Endo G release from the mitochondria to the cytosol [35]. In the present study, we assessed expression changes in cyt-c, Smac/Diablo (referred to hereafter as Smac), HtrA2/Omi (referred to hereafter as HtrA2) and their downstream proteins, including XIAP, apaf-1, PARP and caspase-3, 6, 7 and 9 via qRT-PCR analysis.

**Figure 3:**
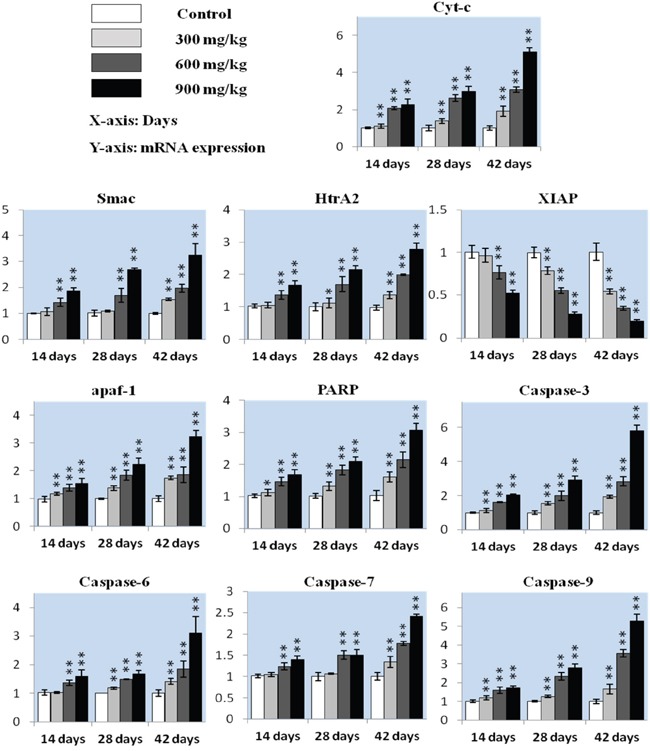
Cyt-c, Smac, HtrA2, XIAP, apaf-1, PARP caspase-3, caspase-6, caspase-7 and caspase-9 mRNA in the kidney Data are presented as means ± standard deviation (n=5). *P<0.05, **P<0.01 compared with the control group.

Cyt-c expression increased (P<0.05 or P<0.01) in the three NiCl_2_-treated groups from 14 to 42 d compared to controls (Figure 3). Smac and HtrA2 levels were higher (P<0.05 or P<0.01) in the 600 mg/kg and 900 mg/kg groups from 14 to 28 d and in the three NiCl_2_-treated groups at 42 d. HtrA2 expression increased (P<0.05) in the 300 mg/kg groups at 28 d. XIAP expression decreased (P<0.05) in the 600 mg/kg and 900 mg/kg groups at 14 d and in the three NiCl_2_-treated groups from 28 to 42 d. Apaf-1 and PARP levels were higher (P<0.05 or P<0.01) in the three NiCl_2_-treated groups from 14 to 42 d.

Caspase-3 expression increased (P<0.05 or P<0.01) in the three NiCl_2_-treated groups from 14 to 42 d. Caspase-6 and caspase-7 levels were higher (P<0.05 or P<0.01) in the 600 mg/kg and 900 mg/kg groups from 14 to 28 d and in the three NiCl_2_-treated groups at 42 d compared to controls. Caspase-6 expression increased (P<0.05) in the 300 mg/kg groups at 28 d, and caspase-9 expression was higher (P<0.05 or P<0.01) in the three NiCl_2_-treated groups from 14 to 42 d.

**Figure 4:**
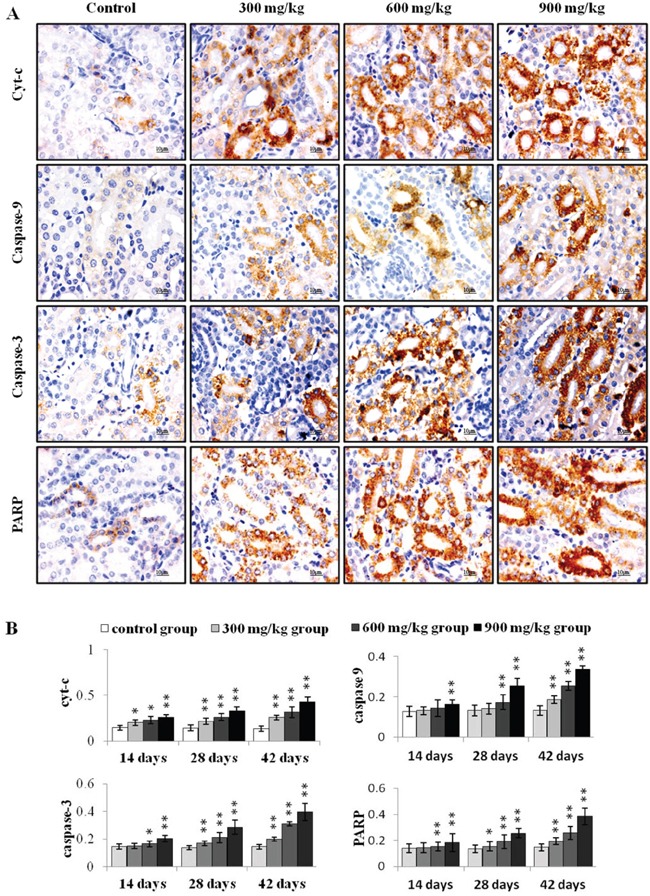
Immunohistochemical analysis of cyt-c, caspase-9, caspase-3 and PARP in the kidney **A.** Representative immunohistochemical staining results for cyt-c, caspase-9, caspase-3 and PARP in the kidney at 42 d. **B.** Quantification of cyt-c, caspase-9, caspase-3 and PARP protein levels in the kidney. Data are presented as means ± standard deviation (n=5×5). *P<0.05, **P<0.01 compared with the control group.

Cyt-c, caspase-9, caspase-3 and PARP protein levels were assessed via immunohistochemical staining. Cyt-c protein levels increased (P<0.05 or P<0.01) in the three NiCl_2_-treated groups from 14 to 42 d (Figure 4). Caspase-9 protein was higher (P<0.01) in the 900 mg/kg group at 14 d, in the 600 and 900 mg/kg groups at 28 d, and in the three NiCl_2_-treated groups at 42 d compared to controls. Caspase-3 and PARP protein levels increased (P<0.05 or P<0.01) in the 600 and 900 mg/kg groups at 14 d and in the three NiCl_2_-treated groups from 28 to 42 d.

### Fas-mediated caspase-dependent apoptosis

**Figure 5:**
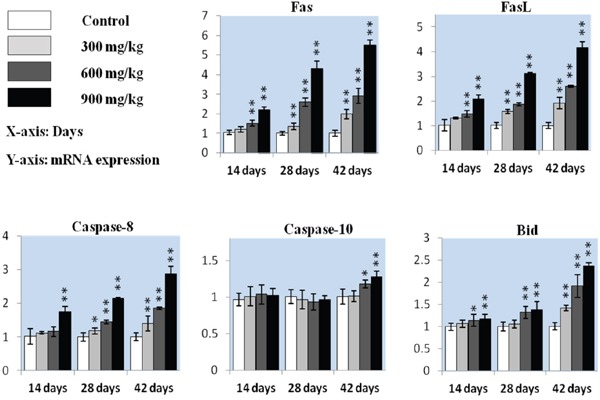
Fas, FasL, caspase-8, caspase-10 and Bid mRNA in the kidney Data are presented as means ± standard deviation (n=5). *P<0.05, **P<0.01 compared with the control group.

We measured whether the Fas-mediated caspase-dependent apoptotic pathway played a role in NiCl_2_-induced apoptosis. Fas and FasL mRNA levels were increased (P<0.05 or P<0.01) in the 600 mg/kg and 900 mg/kg groups from 14 to 28 d and in the three NiCl_2_-treated groups from 14 to 42 d compared with controls (Figure 5). Caspase-8 expression was higher (P<0.05 or P<0.01) in the 900 mg/kg groups at 14 d and in the three NiCl_2_-treated groups from 28 to 42 d. Caspase-10 expression increased (P<0.05 or P<0.01) in the 600 and 900 mg/kg groups at 42 d. Bid expression was higher (P<0.05 or P<0.01) in the 600 mg/kg and 900 mg/kg groups from 14 to 42 d and in the 300 mg/kg groups at 42 d.

**Figure 6:**
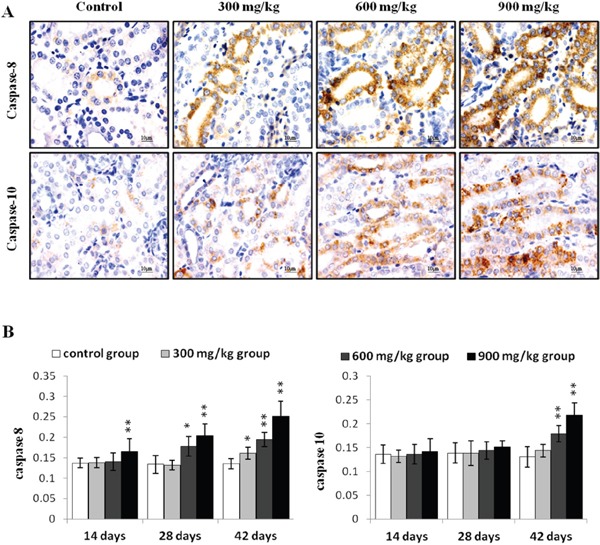
Immunohistochemical analysis of caspase-8 and caspase-10 in the kidney **A.** Representative immunohistochemical staining results for caspase-8 and caspase-10 in the kidney at 42 d. **B.** Quantification of caspase-8 and caspase-10 protein levels in the kidney. Data are presented as means ± standard deviation (n=5×5). *P<0.05, **P<0.01 compared with the control group.

Caspase 8 protein levels were higher (P<0.05 or P<0.01) in the 900 mg/kg group at 14 d, in the 600 and 900 mg/kg groups at 28 d, and in the three NiCl_2_-treated groups at 42 d compared to controls (Figure 6). Caspase 10 protein increased (P<0.05 or P<0.01) in the 600 and 900 mg/kg groups at 42 d.

### Ni residue in the kidney

Renal Ni accumulation was greater (P<0.05 or P<0.01) in the three NiCl_2_-treated groups at 42 d compared to controls [20].

## DISCUSSION

In this study, we found that NiCl_2_ induced time- and dose-dependent apoptosis and functional injury in the kidney. Histopathological lesions, functional damage and apoptosis are consistent with Ni accumulation, indicating that Ni accumulation is a direct cause of renal injury. In our previous studies, TUNEL and histopathological results showed that NiCl_2_ induces apoptosis in the kidney [20, 21], thymus, spleen and cecal tonsil [17, 25, 36]. Our results are consist with those of Zheng, *et al.* [37] who showed that NiSO_4_ induces JNK-mediated oxidative stress and apoptosis in *Carassius auratus* liver. Ni compounds can increase apoptosis in HepG2 cells [38], normal rat kidney cells [39], and human neutrophils and lymphocytes [40, 41]. Based on these and other findings, a number of groups have focused on the potential intrinsic and extrinsic apoptotic signaling pathways induced by Ni and Ni compounds.

In the intrinsic apoptosis pathway, e.g., the mitochondria-mediated caspase-dependent apoptotic pathway, the mitochondria plays a key role in apoptosis [42]. The death signal disrupts the mitochondrial membrane and pro-apoptotic proteins, including cyt-c, Smac, HtrA2, AIF and Endo G, are released from the mitochondria into the cytosol [43–47]. We previously found that NiCl_2_ induces mitochondrial-mediated caspase-independent apoptosis via MMP damage and increased AIF and EndoG expression [35]. Our present study showed that cyt-c, Smac and HtrA2 levels were also increased after NiCl_2_-induced MMP disruption [21]. Zhao, *et al.* [33] reported that cyt-c and AIF release from the mitochondria into the cytoplasm increased after exposure to NiNPs and Ni fine particles. Cyt-c can activate the caspase-dependent apoptosis pathway [44].

The results of this study showed that NiCl_2_ increased apaf-1, caspase-9, caspase-3, caspase-6, caspase-7 and PARP expression and decreased XIAP levels. Increased transcription of these genes is the most probable explanation for activation of mitochondria-mediated caspase-dependent apoptosis. NiCl_2_ also increased cyt-c, caspase-9, caspase-3 and PARP protein levels. NiNPs and Ni fine particles reportedly increase caspase-3, 6 and 9 protein levels and activation in JB8 and A431 cells [33, 48]. Nickel ferrite nanoparticles increase caspase-3 and caspase-9 activation and expression in HepG2 and MFC-7 cells [49], and NiSO_4_ increases hepatic caspase-3 activity in *Carassius auratus* [37]. Patel, *et al.* [50] suggested that NiCl_2_ increases caspae-3 and caspase-7 protein levels in human lung epithelial cells. Caspase-3 activity also increases in NiNP-treated cells in a dose- and time-dependent fashion [51]. NiONPs increase numbers of Annexin V positive cells and caspase-3 activation [30]. NiCl_2_ treatment promotes MMP disruption and cyt-c release into the cytosol, which in turn cleaves and activates caspase-9 [44]. Activated caspase-9 can cleave and activate caspase-3, 6 and 7, inducing PARP cleavage and apoptosis [52, 53]. XIAP suppresses apoptosis by directly inhibiting caspase-3 and -9 [54, 55]. Smac and HtrA2 reportedly promote apoptosis by inhibiting IAP activity [56]. Increased Smac and HtrA2 and decreased XIAP expression also contribute to apoptosis.

**Figure 7:**
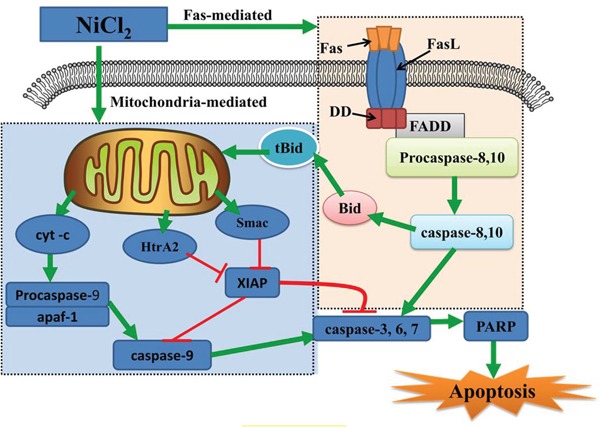
Schematic diagram of NiCl_2_-induced mitochondria- and Fas-mediated caspase-dependent apoptosis NiCl_2_ increases the cyt-c, Smac and HtrA2 release from the mitochondria into the cytosol. Cyt-c cleaves and activates caspase-9, which in turn cleaves and activates downstream caspases, such as caspase-3, 6 and 7. Caspase-3, 6 and 7 cleave PARP, which then induces apoptosis. Concurrently, Smac and HtrA2 inhibit XIAP expression, also contributing to apoptosis. NiCl_2_ also promotes Fas and Fas ligand interactions, leading to activation of caspase-8 and casapase-10. Activated caspase-8 and 10 can directly cleave and activate downstream effector proteases, such as caspase-3, 6 and 7, leading to apoptosis.

In this study, increased Fas, FasL, caspase-8, caspase-10 and Bid transcription was the most probable explanation for the activation of Fas-mediated (extrinsic) caspase-dependent apoptosis. Our results showed that NiCl_2_ increased caspase-8 and caspase-10 protein levels, two important components of the Fas-mediated caspase-dependent apoptosis pathway. In this pathway, FasL combined with the Fas receptor to activate caspase-8 and 10 [57]. Activated caspase-8 and 10 could directly cleave and activate caspase-3, 6 and 7, leading to apoptosis [58]. Our results are consistent with those of Zhao, *et al.* [33] who found that metallic Ni particles increased Fas, FADD and caspase-8 expression in JB6 cells. Moreover, caspase-8 and caspase-10 can also cleave the Bcl-2 family member Bid to tBid. tBid can bind to Bax, inducing MMP disruption and cyt-c release [59]. Therefore, NiCl_2_-medicated Bid cleavage may create a crucial connection between intrinsic and extrinsic apoptosis. In Figure 7, we summarize the possible mechanism of NiCl_2_-induced tubular apoptosis via mitochondria- and Fas-mediated caspase-dependent apoptotic pathways.

In conclusion, the present study showed that dietary NiCl_2_ at 300+ mg/kg induces tubular apoptosis in broiler chickens, involving both mitochondria- and Fas-mediated caspase-dependent apoptotic pathways. Our results provide novel insights into Ni and Ni compound toxicology *in vitro* and in the broiler chicken kidney *in vivo*.

## MATERIALS AND METHODS

### Experimental design

Two hundred and eighty one-day-old healthy broiler chickens (Chia Tai Group, Wenjiang, Sichuan, China) were divided into four groups (N=70). All experimental procedures involving broiler chickens were approved by Animal Care and Use Committee, Sichuan Agricultural University (Approval No: 2012-024). Chickens were housed in cages with electrical heaters and provided with feed and water, as well as the experimental diets, *ad libitum* for 42 d. A corn-soybean meal formulated by the National Research Council [60] was the control diet, and NiCl_2_ (NiCl_2_·6H_2_O, Cheng Du Kelong Chemical Co., Ltd., Chengdu, China) was mixed into this basal diet to produce experimental diets containing 300, 600 or 900 mg/kg NiCl_2_. These three doses were chosen based on the results of previous studies. Ling and Leach reported that dietary NiCl_2_ concentrations of 300 mg/kg or more resulted in reduced growth rates. Mortality and anemia were observed in chicks receiving 1100 mg/kg Ni [61]. Weber and Reid observed a growth reduction at 700 mg/kg or more NiSO_4_ or nickel acetate [62]. Chicks fed more than 250–300 mg/kg Ni exhibited depressed growth and reduced feed intake [63]. Bersenyi, *et al.* [64] reported that supplementation with 500 mg/kg NiCl_2_ reduced weight gain by 10% and feed intake by 4%, and reduced feed conversion efficiencies by 5% in growing broiler cockerels.

### Macroscopic kidney examination

At 14, 28 and 42 d, five chickens in each group were euthanized and necropsied. Kidneys were observed and weighed after dissecting connective tissue around the organ. Relative kidney weight was calculated using the following formula: Relative weight = organ weight (g)/body weight (kg).

### Clinical pathological kidney examination

At 14, 28 and 42 d, five broiler chickens in each group were phlebotomized from the jugular vein to collect serum. Non-anticoagulative blood samples were clotted for 15 min at room temperature and then centrifuged at 3000 rpm for 15 min. Serum creatinine and uric acid were detected by biochemical methods following the manufacturer's instructions (creatinine, C011-1; uric acid, C012-1; Nanjing Jiancheng Bioengineering Institute of China, Nanjing, China).

### Histopathological kidney examination

Histopathological examination of the kidney was performed as previously described [20].

### Apoptosis analysis by flow cytometry

At 14, 28 and 42 d, five broilers in each group were used to assess apoptosis in the kidney by flow cytometry as described by Tang, *et al.* [17]. Briefly, broilers in each subsample were humanely killed, and kidneys were immediately ground to form a cell suspension, which was filtered through a 300-mesh nylon screen. Cells were washed twice with ice-cold PBS (pH 7.2–7.4), and then suspended in PBS at 1×10^6^ cells/mL. A total of 100 μL of the cell suspension was transferred to a 5-mL culture tube. Cells were stained with 5 μL Annexin V-FITC (Cat: 51-65874X, BD, USA) and 5 μL of PI (Cat: 51-66211E, BD, USA) at 25°C for 15 min in the dark. Finally, 400 μL of 1× binding buffer was added to each tube and cells were analyzed by flow cytometry (BD FACSCalibur) within 1 h of preparation. Results were analyzed using the Mod Fit LT for Mac V3.0 program.

### Quantitative real-time PCR

Kidneys were taken at 14, 28 and 42 d from five broilers in each group and stored in liquid nitrogen. They were then homogenized in liquid nitrogen using a mortar and pestle. Total RNA was isolated using RNAiso Plus (9108/9109, Takara, Japan). RNA was reverse transcribed to cDNA using the Prim-Script™ RT reagent Kit (RR047A, Takara, Japan) according to the manufacturer's protocol. cDNA was used as a template for qRT-PCR analysis. Sequences for target apoptosis-related genes were obtained from the NCBI database. Oligonucleotide primers were designed using Primer 5 software and synthesized at Takara (Dalian, China; Table 1).

**Table 1:** qRT-PCR primers

Gene symbol	Accession number	Primer	Primer sequence (5′-3′)	Product size	Tm (°C)
cyt-c	NM001079478	Forward Reverse	TGTCCAGAAATGTTCCCAGTGC CCTTTGTTCTTATTGGCATCTGTG	138bp	60
XIAP	NM204588	Forward Reverse	CTAAACAACGAACAGCATCCAAGG ACAACGTGATCGCCATTACCTG	146bp	58
HtrA2	XM423666	Forward Reverse	CATCCAGACAGACGCCGCTAT CAGGAACTTTCGCAGTCGGTC	145bp	62
Smac	XM415152	Forward Reverse	TCCCAGAAGGCAGAGACCAAG GGTCCTCACCCGCATCTGTA	118bp	62
apaf1	XM416167	Forward Reverse	AAGGGCATAAGGAAGCAATCAA CAGCACAAGAAAGAACAGCACC	156bp	61
caspase-9	AY057940	Forward Reverse	CGAAGGAGCAAGCACGACAG CCGCAGCCCTCATCTAGCAT	130bp	61
caspase-3	NM204725	Forward Reverse	TGGCCCTCTTGAACTGAAAG TCCACTGTCTGCTTCAATACC	139bp	62
caspase-6	AF469049	Forward Reverse	TCAGAGGAGACAAGTGCCAGAGT TACTGAATCCTGAACGAGAACTGG	107bp	59
caspase-7	XM421764	Forward Reverse	CCGAAGTCCTCACTCAGTAACCA TTGCGTGTACCCATTCCTGTT	137bp	58
Fas	NM001199487	Forward Reverse	TGTTCGTCATCACCGTCTATCG TTCGTAGGCTCCTCCCATCC	133bp	60
FasL	AJ890143	Forward Reverse	AGATCGCATCCCTCCAGCTC GAGACAGGTTCCCACTCCAATG	135bp	59
caspase-8	NM204592	Forward Reverse	TGGGAAAGTGGACAAGAGCCT CCACAGATGATGCCAGCCAA	146bp	59
caspase-10	XM421936	Forward Reverse	GCAGCGTTCAGAAGACCACAA CATTGCTTGGCAGTGAAGTAGGT	141bp	61
Bid	NM204522	Forward Reverse	AGTGGAAGGACTTGCCAGAGC TTGTGGAAGTGTTGGCTGATGTA	162bp	60
PARP	NM205263	Forward Reverse	AAGCTCCGAACTGATATTAAGGTGG GCTTAAATGGCTTGTAACGCTGA	172bp	56
β-actin	L08165	Forward Reverse	TGCTGTGTTCCCATCTATCG TTGGTGACAATACCGTGTTCA	178bp	62

qRT-PCR reactions (25 ul each) included 12.5 ul SYBR^®^ Premix Ex Taq™II (DRR820A, Takara, Japan), 1 ul forward and 1 ul reverse primer, 8.5 ul of RNAase-free water (RT12102, Tiangen, China) and 1 ul of cDNA. A Bio Rad C1000 Thermal Cycler (Bio Rad, USA) was used to perform qRT-PCR reactions. The PCR procedure consisted of 95°C for 3 min followed by 44 cycles of 95°C for 10 s, Tm of a specific primer pair for 30 s, and then 95°C for 10 s, 72°C for 10 s. Melting curve analysis showed only one peak for each PCR product.

Chicken β-actin was used as an internal reference housekeeping gene. Gene expression values from control group subsamples at 14, 28 and 42 d were used to calibrate gene expression in experimental subsamples. Expression fold changes were calculated using the 2^−ΔΔCT^ method [65].

### Immunohistochemistry

Five broilers in each group were humanely sacrificed for gross examination at 14, 28 and 42 d. Kidneys were collected and fixed in 4% paraformaldehyde, dehydrated in ethanol and embedded in paraffin. As described by Wu, *et al.* [25], renal slices were dewaxed in xylene, rehydrated through a graded series of ethanol solutions and washed in distilled water and PBS. Endogenous peroxidase activity was blocked by incubation with 3% H_2_O_2_ in methanol for 15 min. Slices were subjected to antigen retrieval by microwaving in 0.01 M sodium citrate buffer pH 6.0. Additional washing in PBS was performed before 30 min of incubation at 37°C in 10% normal goat serum (Boster, Wuhang, China). Slices were incubated overnight at 4°C with caspase-9 (1:100) antibody, which could recognize both pro- and cleaved-caspase-9 (ab32539, Abcam, USA), cleaved-caspase-3 (1:2000) antibody (9664, CST, USA), cleaved-caspase-8 (1:100) antibody (9496, CST, USA), caspase-10 (1:100) antibody, which could recognize both the pro- and cleaved-caspase-10 (sc-6184, Santa Cruz, USA), cyt-c (1:100) antibody (sc-13560, Santa Cruz, USA) or cleaved-PARP (1:50) antibody (5625, CST, USA). After washing in PBS, slices were exposed to 1% biotinylated goat anti-mouse IgG secondary antibody (Boster, Wuhang, China) for 1 h at 37°C, and then incubated with streptavidin-biotin complex (SABC; Boster, Wuhang, China) for 30 min at 37°C. To visualize the immunoreaction, slices were immersed in diaminobenzidine hydrochloride (DAB; Boster, Wuhang, China). Slices were monitored microscopically and stopped by immersion in distilled water as soon as brown staining was visible. Slices were lightly counterstained with hematoxylin, dehydrated in ethanol, cleared in xylene and mounted.

Protein expression was quantitatively assessed using a computer-supported imaging system connected to a light microscope (Olympus, Shimadzu, Japan) with an objective magnification of ×1000. Staining intensity for each protein was quantified using Image-pro Plus 5.1 (Madia Cybernetics, MD, USA). Five sections were measured from each group and five fields from each section were measured and averaged [25].

### Renal Ni quantification by GFAAS

Five broilers in each group were humanely killed at 42 d, and kidneys were immediately removed, weighed, dried and collected for determination of Ni. Ni concentrations in the kidney were measured by GFAAS as previously described [20].

### Statistical analysis

All treatment groups were compared to their respective controls. Significant differences among the three treatment groups and the control group were analyzed by one-way ANOVA. Results are presented as means ± standard deviation (M ± SD). All tests were performed using SPSS 16.0 for Windows.

### Abbreviations

Abbreviations appeared in the text are listed in the Table 2.

**Table 2:** Abbreviations appeared in the text

Abbreviation	Name	Abbreviation	Name
Ni	Nickel	MMP	mitochondrial membrane potential
NiCl_2_	Nickel Chloride	Cr	creatinine
qRT-PCR	Quantitative Real Time-polymerase Chain Reaction	UA	uric acid
cytochrome c	cyt-c	PS	phosphatidylserine
HtrA2/Omi	High-temperature-requirement protein A2	AIF	apoptosis inducing factor
Smac/Diablo	second mitochondria-derived activator of caspase	Endo G	endonuclease G
apaf-1	apoptotic peptidase activating factor 1	TUNEL	terminal deoxynucleotidyl transferase 2'-deoxyuridine 5′-triphosphate dUTP nick end-labeling
PARP	poly (ADP-ribose) polymerase	JNK	c-Jun N-terminal kinase
XIAP	X-linked inhibitor of apoptosis protein	HepG2	human hepatocellular carcinoma
FasL	Fas ligand	PI3K	phosphoinositide-3-kinase
Bid	BH3 interacting domain death agonist	Akt	serine-threonine kinase
NiSO_4_	Nickel Sulfate	NiNPs	nickel nanoparticles
IARC	International Agency for Research on Cancer	FCE	feed conversion efficiency
NiONPs	NiO nanoparticles	PI	propidium iodide
Ni(tcitr)_2_	bis (S-citronellalthiosemicarbazonato) Ni (II)	GFAAS	Graphite Furnace Atomic Absorption Spectrometry
